# Two-Year Outcomes Using Fast-Acting, Sub-Perception Therapy for Spinal Cord Stimulation: A European, Real-World, Multicenter Experience

**DOI:** 10.3390/jcm13226999

**Published:** 2024-11-20

**Authors:** Simon Bayerl, Jose Paz-Solis, Georgios Matis, Philippe Rigoard, Jan Willem Kallewaard, M. Angeles Canos-Verdecho, Jan Vesper, Jose Emilio Llopis, Georgios Kyriakopoulos, Ashish Gulve, Sylvie Raoul, Alfonso Papa, Sarah Love-Jones, Adam Williams

**Affiliations:** 1 Inter Neuro Berlin, 10629 Berlin, Germany; 2 Department of Neurosurgery, Charite University Hospital, 10117 Berlin, Germany; 3 Department of Neurosurgery, Hospital Universitario La Paz, 28046 Madrid, Spain; pazsolis@icloud.com; 4 Department of Stereotactic and Functional Neurosurgery, Uniklinik Köln, 50937 Köln, Germany; georgios.matis@uk-koeln.de; 5 Department of Neuro-Spine and Neuromodulation, Poitiers University Hospital, 86021 Poitiers, France; 6 Department of Anesthesiology and Pain Management, Rijnstate Hospital, 6815 AD Arnhem, The Netherlands; jkallewaard@rijnstate.nl; 7 Department of Anesthesiology and Pain Management, Hospital Universitario y Politécnico La Fe, 46026 Valencia, Spain; angelescanos@hotmail.com; 8 Department of Functional Neurosurgery and Stereotaxy, Universitaetsklinikum Heinrich-Heine, 40225 Düsseldorf, Germany; 9 Department of Anesthesiology and Pain Management, Hospital de la Ribera, 46600 Valencia, Spain; 10 Johanniter-Kliniken Hamm, 59063 Hamm, Germany; 11 Department of Anesthesiology and Pain Management, James Cook University Hospital, Middlesbrough TS4 3BW, UK; 12 Department of Neurosurgery, Centre Hospitalier Universitaire Laennec, 44800 Nantes, France; 13 Department of Anesthesiology and Pain Management, A.O. Dei Colli -V. Monaldi Hospital, 80131 Napoli, Italy; 14 Anesthesiology, Pain Management and Neurosurgery, Southmead Hospital, Bristol BS10 5NB, UK

**Keywords:** chronic pain, fast-acting, spinal cord stimulation, sub-perception, surround inhibition

## Abstract

**Background/Objectives:** Over the last 20 years, spinal cord stimulation (SCS) has seen the development of various paresthesia-free paradigms. Recently, a novel modality has emerged (Fast-Acting Sub-perception Therapy, FAST) that engages the surrounding inhibition mechanism of action. We evaluated long-term, real-world outcomes of preferential FAST-SCS use in patients with chronic pain. **Methods:** In this multi-center, observational, consecutive case series, medical chart data from chronic pain patients preferentially using FAST-SCS (no exclusions) were retrospectively reviewed. **Results:** Data from 167 patients in 13 European centers were analyzed; 74% of patients suffered from persistent spine pain syndrome type 2 and 87% presented with low back and/or leg pain. At the last follow-up (mean 1.6 years), the numerical rating scale (NRS) overall pain score decreased by 5.1 ± 2.5 points versus baseline, from 8.0 ± 1.2 to 2.9 ± 2.2 (n = 167, *p* < 0.0001). 87% of patients reported ≥50% pain relief, and 55% were “high responders” with overall NRS pain scores ≤2/10. At the last follow-up, functional disability improved significantly (the Oswestry Disability Index reduced by 29.2 ± 21.5 points, n = 65, *p* < 0.0001) and patients had a significant gain in quality of life (EQ-5D-5L visual analog scale increased by 52.0 ± 26.9 points, n = 86, *p* < 0.0001). Results at the 2-year follow-up showed a sustained, substantial reduction in pain; 67% of patients were high responders and the NRS overall pain score decreased by 5.6 ± 2.4 versus baseline (n = 52, *p* < 0.0001). **Conclusions:** Our real-world outcomes suggest that in patients with chronic low back and/or leg pain, FAST-SCS therapy provided durable and profound pain relief and led to significant improvements in disability and quality of life.

## 1. Introduction

Spinal cord stimulation (SCS) has been used to manage pain since the late 1960s after Melzack and Wall developed their gate control theory of pain [1]. The theory states that inputs from nociceptive and non-nociceptive primary fibers (such as Aβ mechanoreceptors) are filtered by the complex spinal inhibitory interneuron circuits (the “gate”), which controls how strongly the painful signals will be transmitted to higher brain centers. In the context of neuropathic pain, abnormal processing of somatosensory input leads to a decrease in synaptic inhibition in the dorsal horn (disinhibition), allowing nociceptive information to be transmitted.

Spinal cord stimulation was hypothesized to produce an analgesic effect in patients with neuropathic pain by elevating the activity of non-nociceptive sensory fibers corresponding to the pain area [2,3]. Electrical stimulation applied to the dorsal column fibers also produced a “tingling” sensation, described as paresthesia. For optimal pain relief, the goal of SCS device programming was to ensure that paresthesia coverage overlapped with the painful areas [2,3].

As a result, paresthesia-based SCS programming (or programming above sensory perception) using low-frequency settings (40–100 Hz) has predominantly been used in clinical practice since the beginning of SCS therapy [4]. Paresthesia-based SCS therapy is still in use today and has benefited from various technological evolutions over time (current control, multi-contact lead design, electrical field steering, algorithmic programming, amplitude adjustments, etc.), enhancing precise targeting and stable activation of the dorsal column sensory fibers [5,6,7,8].

In 2006, new SCS settings used by Yearwood [9] did not induce any perception of tingling sensations in patients, marking the beginning of a new SCS paradigm. By engaging different mechanisms of action, such as activation of the medial pathway and/or preferential activation of inhibitory interneurons in the dorsal horn [10,11,12,13,14,15,16,17,18], sub-sensory threshold stimulation waveforms have offered new opportunities for pain management [19,20,21,22,23,24,25,26,27,28,29,30,31]. Shortly after the conventional forms of sub-sensory threshold SCS became available (burst and kilohertz frequency stimulation), new concepts for neural targeting [32] and neural dosing [33] were developed to best engage sub-perception mechanisms while optimizing the efficiency of these paradigms in both spatial and temporal domains.

More recently, a novel, paresthesia-free modality has emerged [34,35] that explores whether paresthesia (an indicator of spinal organization) could be used to target pain in sub-perception SCS [36]. Thus, a new SCS approach was developed to test this hypothesis, where paresthesia was steered at high resolution and at the lowest practical amplitude capable of inducing paresthesia to cover all painful areas [37,38]. Surprisingly, reducing the amplitude below the perception threshold led to most patients reporting profound pain relief within minutes following activation of SCS therapy.

**Figure 1:**
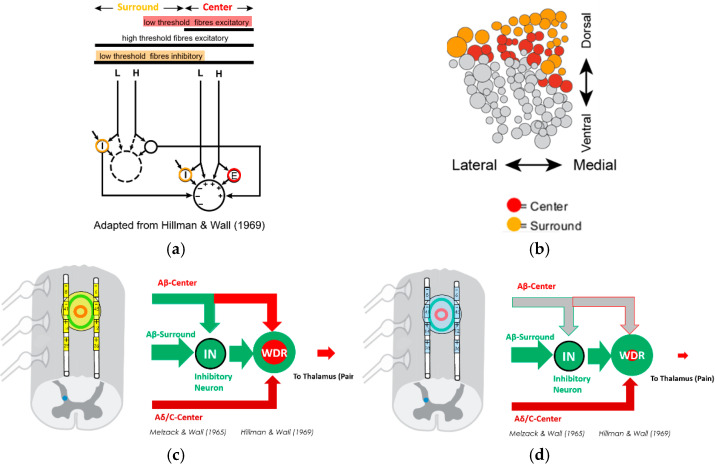
(**a**) Inhibitory and/or excitatory effects of sensory fibers from surround versus center receptive fields [39]; (**b**) topography of dorsal column axons under optimal targeting conditions; (**c**) mixed effects of paresthesia-based spinal cord stimulation (SCS) (supra-perception intensity) with the gate control theory mechanism [1,39]; (**d**) inhibitory effects of Fast-Acting Sub-perception Therapy (FAST)-SCS (sub-perception intensity) with the surround inhibition mechanism [39].

This novel, sub-sensory threshold approach—Fast-Acting Sub-perception Therapy (FAST™)—utilizes the surround inhibition mechanism, mediated by spinal cord dorsal columns. In fact, as a further understanding of the gate control theory, the disinhibition produced by a nerve injury increases the excitability and activation of sensory pathways that are normally silenced by inhibition (Figure 1a,b). In contrast to paresthesia-based SCS, which primarily targets the center sensory receptive field fibers to restore the inhibitory function of the gate (Figure 1c), FAST engages the surround inhibition mechanism to further reduce the excitatory inputs responsible for chronic pain (Figure 1d) [39].

FAST also presents additional interesting characteristics compared to other sub-sensory threshold SCS modalities, such as the possibility of using a more efficient low-frequency stimulation pattern and the rapid onset of analgesia minutes after therapy activation. This contrasts with the traditional slow onset of analgesia and high-demand energy patterns typically observed for other paresthesia-free SCS modalities (hours to days) [34].

Robust pre-clinical and computational modelling research studies, including recording of dorsal column axons and dorsal horn neurons, have been conducted and led to a better understanding of how low-frequency and low-amplitude SCS could produce pain relief via a novel mechanism (surround inhibition) [35].

In 2021, Metzger et al. [34] assessed the onset of pain relief after FAST activation in a cohort of 41 patients. This study showed that a few minutes after the novel sub-perception modality was turned “ON”, patients perceived profound pain relief, with the average pain score decreasing from 6.5/10 in the stimulation “OFF” state to 1.3/10, on average 11 min after FAST-SCS was activated. These clinical observations objectivized and confirmed the fast-acting behavior of this new modality, contrasting with other sub-perception modalities. It also confirmed the maintenance of low pain scores at up to 6 months of follow-up; however, it lacked assessment of FAST-SCS clinical outcomes in a larger patient population, and with longer-term data.

Our multicenter, European study retrospectively investigated the real-world clinical outcomes of SCS patients who reported using FAST therapy as their preferred SCS program. We hypothesized that patients who used this novel sub-perception SCS modality might report significant improvements in their pain scores, functional outcomes, and quality of life, which would be sustained long term.

## 2. Materials and Methods

### 2.1. Study Design

The primary objective of this Boston Scientific-sponsored study (ClinicalTrials.gov identifier NCT01550575, accessed on 18 November 2024) was to characterize real-world clinical outcomes with the use of SCS systems for the treatment of chronic pain, via a retrospective review of medical charts. The study included multiple independent cohorts to evaluate outcomes in different subgroups. The cohort presented here is the related to preferential use of FAST-SCS therapy at the time of the latest follow-up. Data was obtained from de-identified patient records in a consecutive case series performed in 13 centers throughout Europe; initial results are presented here. Each site obtained Ethics Committee approval for the study, which was conducted in accordance with Good Clinical Practice (ISO14155) guidelines [40] and the Declaration of Helsinki. All patients provided written, informed consent as per local regulatory requirements.

### 2.2. Study Setting and Participants

There were no exclusion criteria, as per the study protocol. All patients eligible for SCS whose indications were compliant with the “directions for use” labeling of the new device and with local regulations were included in the study.

Consecutive chronic pain patients (aged ≥18 years at the time of the baseline) who had been implanted with a multimodal SCS system (Boston Scientific Spinal Cord Stimulation Systems) and had reported preferential use of FAST therapy during their most recent follow-up visits were included in this cohort analysis.

Each center applied their standard practice to decide which modalities patients were exposed to during the SCS trial as well as after implantation of the pulse generator. Only patients using FAST therapy as a preferred treatment at the time of the follow-up clinical evaluations were included in the analysis. Patients who were given the possibility of using another SCS therapeutic option besides FAST were not excluded from the study.

### 2.3. Fast-Acting Sub-Perception Therapy

Standard techniques were used to place the SCS leads, according to the preference of each implanting physician.

The programming of FAST-SCS was consistent across all sites and for all patients. Using paresthesia as an indicator of spinal organization, the first programming step was to use supra-perception stimulation as a marker for targeting surrounding receptive field fibers; paresthesia was steered at high resolution, using biphasic, symmetric pulses at 90 Hz frequency, 210 ± 50 µs pulse width, and at the lowest practical amplitude that could induce paresthesia overall painful areas [5,32]. This novel stimulation pattern was characterized by the implementation of two separate central points of stimulation (CPS)—one for each rectangular phase of the charge-balanced stimulation cycle. In the first rectangular phase, negative current is injected through the “cathodes” (negatively configured contacts) and positive current through the “anodes” (positively configured return contacts). In the second rectangular phase, charge balance is achieved by reversing the polarities (i.e., the current becomes anodic at the assigned cathode and vice versa); thus, a second CPS is implemented in the anodic region.

A neural target search using two CPSs (8–16 mm apart) was carried out to identify the electrode configuration and fine tune the stimulation location. To that effect, and using the “fine” resolution setting (which enables ~300 μm incremental shifts in the neurostimulation field), the CPSs were simultaneously steered in the rostro-caudal and medial-lateral dimensions [14]. This allowed the stimulating field to be systematically optimized to maintain complete overlap between the sensation of paresthesia (at an amplitude just above the perception threshold) and painful areas even under various body postures and common physical activities (e.g., walking, moving limbs).

Once paresthesia coverage had been optimized, FAST therapy was activated by lowering the amplitude to approximately 30–40% of the perception threshold.

### 2.4. Outcome Measures

The sites and their medical staff collected all data, as per standard practice, without sponsor involvement. Patient assessments were made before implantation of the multimodal SCS system and during the follow-up visits. Demographic information, pain location, and surgical history were all recorded. The numerical rating scale (NRS, scored from 0 = no pain to 10 = worst pain) was used to evaluate pain intensity (mild pain, score ≤ 3; moderate pain, score 4–6; severe pain, score ≥ 7) [41]). Where documented, the Oswestry Disability Index (ODI; scale 0–100) was used to evaluate functional disability (the ODI is an index derived from the Oswestry Low Back Pain Questionnaire, used by clinicians and researchers to quantify disability for low back pain). Quality of life used general assessment of overall health (EQ-5D-5L visual analog scale (VAS) score: 0–100). Adverse events were not collected in the study.

Because of the real-world retrospective nature of the study, data collection and analyses were based on the pre-existing documentation of the patients’ clinical assessments, as performed by the sites per their standard of care follow-up practice. As a result, the number of observations for each data point may differ and fluctuate over time. Available data was entered directly into an electronic data collection system by the site personnel, without the involvement of the study sponsor.

### 2.5. Statistical Analysis

The normality of the change in NRS score was confirmed by a Kolmogorov–Smirnov Test. Means and standard deviations were determined for demographic data and NRS scores, while the score distribution was calculated for NRS and Percent Pain Relief. To calculate whether the mean reduction in baseline pain was greater than 0, a paired *t* test with a two-sided 0.05 significance level was used. Continuous variables are presented as mean ± standard deviation, while categorical variables are presented as frequency (percentage). All statistical analyses were performed using SAS System Version 9.3 software or above (SAS Institute Inc., Cary, North Carolina 27513, USA).

## 3. Results

### 3.1. Patient Population

In total, 167 eligible patients (mean age 60.1 ± 12.5 years, 57.5% females) were included in the analysis (Table 1); all were “de novo” SCS cases. 87% of patients (145/167) had percutaneous leads, either with a 16-contact design (n = 80) or octopolar (n = 65), and 3.6% of patients had surgical leads. 47.3% patients had been implanted with 2 SCS leads (79/167). The study was conducted per standard of care and all patients had been implanted with Boston Scientific multimodal neurostimulators. As a result, while most patients were programmed with FAST as their sole modality, others had the capability to adjust their therapy according to their needs and use other programs via their remote control. In all cases (n = 167, 100%), FAST-SCS was reported as a preferred therapy at the time of the patient’s most recent follow-up visit, which took place 1.6 ± 1.3 years after implantation of the SCS system (median follow-up duration 1.2 years). At the time of the analysis, 52 patients had reached a 24-month follow-up evaluation.

At baseline, 87% of patients (145/167) presented with low back and leg pain, and the majority (124/167, 74%) suffered from persistent spine pain syndrome (PSPS) type 2 [37]. History of spine surgeries included discectomy, vertebral fusion, and lumbar decompression procedures. Prior to SCS therapy, patients had severe pain (NRS overall pain score 8.0 ± 1.2, n = 167) and were severely disabled, as measured using the ODI (59.4 ± 14.4, n = 71) (Table 1).

### 3.2. Overall Pain Relief at the Last Follow-Up

At the last follow-up (mean 1.6 years post-implantation), all patients (n = 167, 100%) had reported FAST as a preferred therapy. At this point, 10–20% of patients reported additional waveform preferences besides FAST-SCS: Contour or Microburst sub-perception modality (n = 32; 19%), combination therapy (n = 29; 17%), or standard rate (n = 17; 10%) were noted as an additional preferred program besides FAST-SCS.

**Figure 2:**
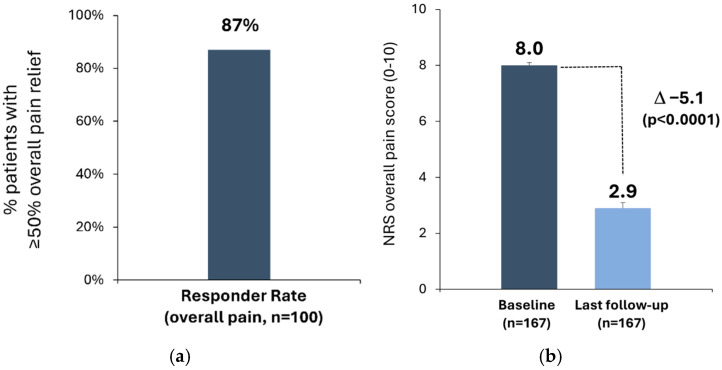
(**a**) Responder rate (% patients who reported ≥50% pain relief) for overall pain at the last follow-up; (**b**) numerical rating scale (NRS) overall pain score (mean ± standard error) at baseline and last follow-up (n = 167). Mean follow-up: 1.6 years after spinal cord stimulation system implantation.

Based on documented assessments, the responder rate was 87% at the last follow-up, at a mean of 1.6 years after SCS implantation (i.e., 87/100 patients reported ≥50% pain relief for their overall pain) (Figure 2a). NRS overall pain score was significantly reduced from 8.0 ± 1.2 to 2.9 ± 2.2 (Δ 5.1 ± 2.5, n = 167; *p* < 0.0001) at last follow-up (Figure 2b).

### 3.3. NRS Pain Score Distribution and High Responders Analysis at the Last Follow-Up

**Figure 3:**
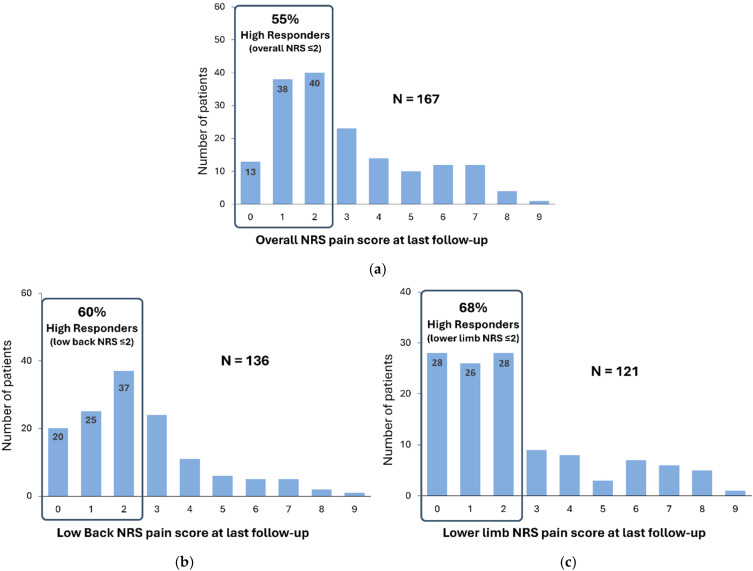
(**a**) Distribution of numerical rating scale (NRS) overall pain scores at the last follow-up (n = 167); (**b**) distribution of NRS low back pain scores at last follow-up (n = 136); (**c**) distribution of NRS lower limb pain scores at last follow-up (n = 121). Mean follow-up: 1.6 years after spinal cord stimulation system implantation.

At baseline, almost of 90% patients presented with low back and leg pain. The distribution of NRS overall pain scores showed that the proportion of “high-responder” patients (subjects who reported NRS pain scores ≤ 2) was 55% (91/167) at the last follow-up, confirming the ability for FAST-SCS to produce profound pain relief (Figure 3a). Results for low back and leg pain were consistent with overall pain: the distribution of NRS low back and leg pain scores showed that 60% (82/136) and 68% (82/121) of patients were “high responders” respectively. At 1.6 years after SCS implantation, patients experienced “mild pain”, with pain scores below 2.5/10 at the last follow-up for both low back and leg pain (2.4 ± 2.0, n = 136 and 2.4 ± 2.4, n = 121, respectively).

### 3.4. Improvements in Low Back Pain and Disability at the Last Follow-Up

Our study population mainly included patients with low back and/or leg pain and had a highly severe disability at baseline as measured by the Oswestry low back pain questionnaire (baseline ODI was 59.7, i.e., nearly at the “crippled” disability level).

**Figure 4:**
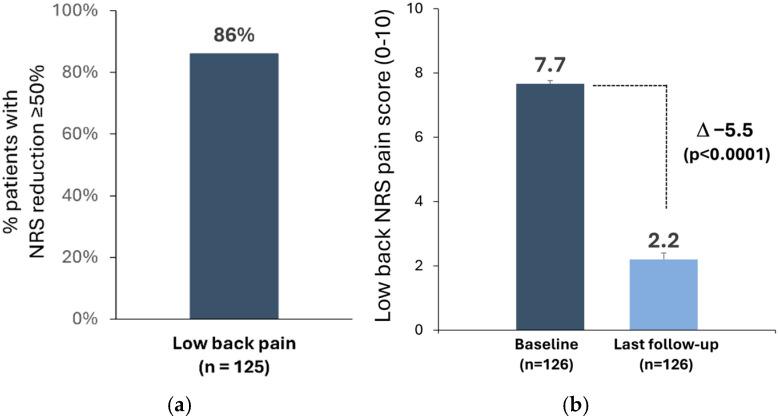
(**a**) Low back pain responder rate (% patients with ≥50% pain reduction) at the last follow-up; (**b**) numerical rating scale (NRS) low back pain score (mean ± standard error) at baseline and last follow-up (n = 126). Mean follow-up: 1.6-years after spinal cord stimulation system implantation.

The low back pain responder rate was 86%; 108/125 patients who had low back pain at baseline reported ≥50% pain relief at the last follow-up (mean 1.6 years) (Figure 4a). The average NRS low back pain score decreased from 7.7 ± 1.8 at baseline to 2.2 ± 1.7 at the last follow-up (Δ −5.5 ± 2.2, n = 126; *p* < 0.0001) (Figure 4b).

**Figure 5:**
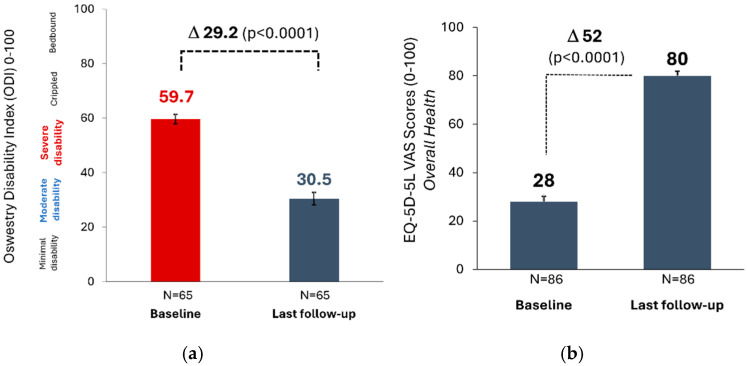
(**a**): Oswestry Disability Index (mean ± standard error) at the last follow-up (n = 65); (**b**): EQ-5D-5L visual analog scale (VAS) score (mean ± standard error) at the last follow-up (n = 86). Mean follow-up: 1.6-years after spinal cord stimulation system implantation.

Functional disability (ODI) was 59.7 ± 13.6 at baseline, corresponding to “severe disability” (40–60). At 1.6 years after SCS implantation, the ODI score decreased to 30.5 ± 18.6 (n = 65), i.e., patients had improved to “moderate” disability. The improvement in ODI compared to baseline was significant (Δ 29.2 ± 21.5; *p* < 0.0001) (Figure 5a)—a change that was almost three times higher than the minimal clinically important difference (MCID) of 10 points for ODI [42,43].

Concordant with the observed outcomes in pain and disability, the overall quality of life in patients improved significantly over the course of their follow-up, with the EQ-5D-5L VAS score increasing by 52.0 ± 26.9 points, from 28.1 ± 19.9 at baseline to 80.1 ± 16.3 at the last follow-up (n = 86; *p* < 0.0001) (Figure 5b).

### 3.5. Long-Term Results at the 2-Year Follow-Up

**Figure 6:**
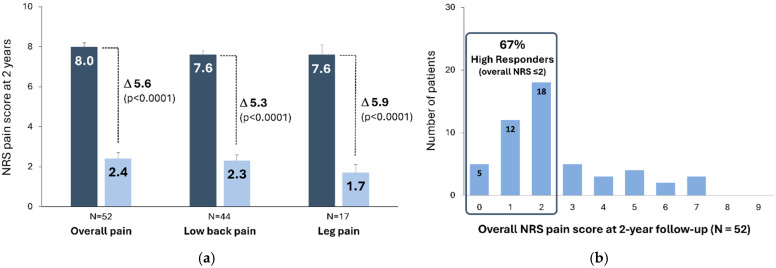
(**a**) 2-year numerical rating scale (NRS) pain scores (mean ± standard error) for overall (n = 52), low back (n = 44) and leg pain (n = 17); (**b**) distribution of overall NRS pain scores at the 2-year follow-up.

Overall, 52 patients had reached the 2-year assessment visits at the time of data analysis. On average, these patients had reported FAST as being a preferred waveform for the last 20 months prior to their 2-year follow-up visit. From their documented outcomes, long-term results showed significant improvements in overall, low back and leg pain. At the 2-year follow-up, the overall pain score was reduced by 5.6 ± 2.4 points (n = 52; *p* < 0.0001). Low back and leg pain improved by 5.3 ± 2.8 points (n = 44; *p* < 0.0001) and 5.9 ± 2.9 points (n = 17; *p* < 0.0001) respectively (Figure 6a). Overall pain as well as low back and leg pain scores were ≤2.5/10 and the distribution of NRS overall pain scores showed that 35/52 (67.3%) patients were high responders (NRS ≤ 2) (Figure 6b).

## 4. Discussion

Over the last 20 years—and after decades of “paresthesia-based” SCS as the only therapeutic option—multiple “paresthesia-free” SCS therapies have been introduced, supported by robust clinical evidence [17,18,19,20,21,22,23,24,31]. As a result, “multi-modal” SCS devices with new programming capabilities have been designed that allow the use of multiple waveforms; these have helped to personalize SCS therapy, account for patient preferences, and sustain efficacy over a long term [21,25,26,28,29,30,44,45].

Further developments in SCS sub-perception modalities have involved the elucidation of specific mechanisms of action [10,11,12,13,14,15,16,17], such as the preferential activation of inhibitory networks in the dorsal horn [12,13,14,15,16,30,32], and how SCS therapy could combine multiple mechanisms to more effectively treat patients with diverse pain profiles [16,29]. New neuromodulation concepts have emerged (e.g., neural targeting and neural dosing) that helped to optimize the efficiency and sustainability of sub-perception SCS outcomes [32,33]. Personalization of SCS therapy has also improved, as patients are now able to proactively manage their individual SCS treatment according to their needs, preferences, and pain dynamic patterns [21,25,26,29,45].

With the discovery of a novel sub-perception paradigm—using precise paresthesia-guided targeting, biphasic pulses, and an optimized neural dose—the “surround inhibition” mechanism [35], mediated by the dorsal columns, can now be applied to the neurostimulative therapeutic field as per standard practice.

Our multicenter, observational, real-world consecutive care series demonstrated that patients who preferentially used FAST-SCS reported a significant and profound reduction in pain scores that were sustained for a mean follow-up of 1.6 years post-SCS implantation. Along with pain relief, this study also showed significant improvements in multiple other important domains such as functional disability and quality of life. Our results are consistent with previous reports [34], and complete initial experiences with longer term real-world data as 67% of patients were high responders (NRS ≤ 2) at the 2-year follow-up.

FAST-SCS is mediated by dorsal column neural elements (Aβ fibers) and uses a standard frequency stimulation pattern, just like conventional paresthesia-based modalities. However, the results of our study suggest that with selective targeting of surround fibers and limited engagement of center fibers, clinical outcomes may be improved significantly. While the literature has shown responder rates of about 60–70% in PSPS type 2 patients when using conventional paresthesia-based SCS, and overall pain decreased by about 50–60% to pain scores of around 3/10 [3,46], this new sub-perception modality—guided by paresthesia—resulted in more profound pain relief, with two-thirds of patients reporting pain scores of 2/10 or less. It is interesting to consider that these differences in clinical outcomes could be explained by the distinct mechanisms that may be in action within the dorsal column, supported by the gate control theory. While conventional paresthesia-based SCS elicits a combination of both inhibitory and excitatory effects, the mechanism of FAST surround inhibition may eliminate the excitatory effects—potentially explaining our observed improvement in clinical response. While this is an ongoing study that will continue to document real-world, long-term outcomes of FAST in clinical practice, research efforts will also help to precisely assess the correlation between clinical outcomes and mechanisms of action in SCS.

Patients with PSPS type 2 typically present with low back and/or leg pain. When the gate control theory was initially translated into clinical practice using neurostimulation of the dorsal columns, the first generation of paresthesia-based SCS devices achieved significant relief of radicular leg pain; however, improvements in low back pain remained modest [46]. Over the years, developments in SCS technology combined with the introduction of novel sub-perception modalities have helped to significantly improve outcomes for low back pain [3,5,9,19]. In our study, FAST-SCS over 1.6 years led to (a) substantial relief of low back pain, with 60% of patients classed as high responders (NRS ≤ 2); (b) an almost 30-point improvement in the ODI disability score (three times higher than the MCID), moving patients from a highly “severe” to a “moderate” level of functional disability. Our results were consistent across various endpoints (low back NRS and ODI), showing that FAST-SCS therapy significantly improved both the intensity of low back pain and its functional impact. These results suggest that activation of surround inhibition via dorsal column fibers is a highly effective solution for relieving low back pain in PSPS type 2 patients.

In addition to these clinical outcomes, FAST-SCS therapy may offer additional noteworthy advantages in the field of sub-perception SCS. Unlike most other waveforms, FAST should be more efficient as it does not require high frequencies or strong neural doses to effectively relieve pain. These advantages may help to revisit sub-perception SCS paradigms, most of which still rely on high-frequency settings and require patients to use rechargeable devices—i.e., high-energy therapy that requires frequent recharging. Patients can now benefit from FAST, a highly effective and efficient sub-perception waveform that is compatible with both rechargeable and non-rechargeable SCS systems, and which requires less frequent recharging. Further developments in the settings for FAST therapy are ongoing, and more neural dosing strategies (autodose programming) have been introduced and recently used in clinical practice. Another advantage is related to the “on-time” aspect, as conventional sub-perception SCS waveforms typically induce a delay of several hours or days before the patient can perceive the therapeutic effects. With FAST, profound pain relief manifests within minutes [34], which may help to optimize patient management workflow—whether at the SCS trial stage or during follow-up programming sessions. It is now possible for the medical team to apply a sub-perception modality and witness how the patient responds during the consultation visit, optimizing healthcare personnel organization, time, and costs.

Our study is associated with some limitations. Due to its nature, our analysis was limited to those data points that had been recorded in the medical charts, per standard of care. While an advantage of the study design was no interference with how patients were assessed and how data was documented by site personnel, our results must be completed and supported by prospective standardized and systematic clinical evaluations. The study did not enable granular documentation of FAST-SCS usage (to assess, for example, how much time per week or month FAST-SCS was activated), which would require the upload and analysis of the neurostimulator data reports. In the context of real-world and standard of care design, the study did not exclude patients who had been given the possibility to use an additional stimulation modality besides FAST-SCS, and who may not have used FAST continuously as their sole modality. The identification and analysis of this case series were based on the “preferential use” of FAST as reported by study subjects at the time when the clinical outcomes were collected; accordingly, the reported results primarily reflect the effects of FAST-SCS. In future studies, more detailed usage insights may be gathered via the analysis of the neurostimulator information.

The results of this study provide new observational evidence supporting the long-term efficacy and durability of this new SCS sub-perception modality. The FAST programming algorithm is available on all multimodal SCS devices, representing one of several SCS therapeutic options that clinicians can use to personalize SCS to each patient and pain profile. While more long-term data is being continuously documented in this study, future research may also look at evaluating the effects of the surround inhibition mechanism in other conditions (e.g., peripheral neuropathies or complex regional pain syndrome).

## 5. Conclusions

Patients with chronic low back and/or leg pain who had chosen FAST-SCS as their preferred program experienced profound and sustained pain relief, with highly significant improvements in overall, low back and leg pain, as well as functional disability, and quality of life over up to 2-years follow-up. These real-world findings demonstrate that by engaging the surround inhibition mechanism, FAST represents a novel, fast-acting, and personalized subperception approach for effective and efficient SCS in patients with chronic low back and/or leg pain. These results need to be confirmed with further evidence from large, prospective, long-term trials.

## Figures and Tables

**Table 1:** Patient characteristics at baseline (N = 167).

Characteristics	Patients
Sex—Females, n (%)	96 (57.5)
Age (years), mean ± SD	60.1 ± 12.5 (n = 163)
Pain location (multiple areas possible), n (%)	
Low back and leg	145 (87)
Low back	127 (76)
Lower limb	91 (54)
Upper limb	10 (6)
Diagnosis for receiving SCS therapy (may have multiple diagnosis), n (%)	
PSPS Type 2	124 (74.3)
Complex regional pain syndrome	20 (12.0)
PSPS Type 1	10 (6.0)
Prior spine surgeries (may have multiple procedures), n (%)	
Lumbar decompression	53 (32)
Spondylodesis	66 (40)
Discectomy	47 (28)
Overall pain intensity (NRS) mean ± SD	8.0 ± 1.2 (n = 167)
Low back pain intensity (NRS) mean ± SD	7.6 ± 1.8 (n = 131)
Leg pain intensity (NRS) mean ± SD	7.8 ± 2.1 (n = 59)
Oswestry Disability Index mean ± SD	59.4 ± 14.4 (n = 71)
Follow-up duration after SCS implant (years), mean ± SD	1.6 ± 1.3 (n = 167)

NRS, numerical rating scale; PSPS, persistent spinal pain syndrome; SD, standard deviation; SCS, spinal cord stimulation.

## Data Availability

The data, analytic methods, and study materials for this clinical study will be made available to other researchers in accordance with the Boston Scientific Data Sharing Policy: https://www.bostonscientific.com (accessed on 14 February 2024).
